# Microvascular dysfunction determines infarct characteristics in patients with reperfused ST-segment elevation myocardial infarction: The MICROcirculation in Acute Myocardial Infarction (MICRO-AMI) study

**DOI:** 10.1371/journal.pone.0203750

**Published:** 2018-11-13

**Authors:** Elisa McAlindon, Maria Pufulete, Jessica Harris, Chris Lawton, Tom Johnson, Julian Strange, Andreas Baumbach, Chiara Bucciarelli-Ducci

**Affiliations:** 1 Department of Cardiology, Bristol Heart Institute, University Hospitals Bristol NHS Trust, Bristol, United Kingdom; 2 Bristol NIHR Cardiovascular Biomedical Research Centre, University Hospitals Bristol NHS Trust and University of Bristol, Bristol, United Kingdom; 3 Heart and Lung Institute, New Cross Hospital, Wolverhampton, United Kingdom; 4 Barts Heart Centre, William Harvey Research Institute, Queen Mary University London, United Kingdom; University of Messina, ITALY

## Abstract

**Background:**

In patients with reperfused ST-elevation myocardial infarction (STEMI) both invasive and non-invasive assessments of microvascular dysfunction, the index of microcirculatory resistance (IMR), and microvascular obstruction (MVO) by cardiovascular magnetic resonance (CMR), independently predict poor long-term outcomes.

**Aims:**

The aims of this study were to investigate whether an invasive parameter (IMR), assessed at the time of primary percutaneous intervention (PPCI), could predict the extent of MVO in proportion to infarct size (MVO index).

**Methods:**

50 patients presenting with STEMI and TIMI flow ≤ I in the infarct related artery were prospectively recruited to the study, before undergoing PPCI. All patients underwent invasive IMR assessment at maximal hyperaemia using adenosine, and following stent insertion. CMR was performed on day 2 following STEMI, MVO was assessed both on first-pass rest perfusion (early MVO) and in the late gadolinium enhancement (LGE) images (late MVO) along with infarct size. The MVO index was calculated as the ratio of late MVO/infarct size. Differences between IMR quartiles and the MVO index were investigated.

**Results:**

The median IMR was 38.5 (range 9 to 202). The median size of late MVO was 1.9% LV (range 0 to 21.0% LV). IMR predicted late MVO (p<0.01) and as IMR increased, the MVO index increased (r = 0.70, [95% CI 0.53, 0.82], p<0.001). An IMR cut-off of 40 significantly predicted the presence of late MVO on CMR (p<0.001).

**Conclusion:**

IMR measured at the time of PPCI in acutely reperfused STEMI is associated with the presence and severity of infarct damage as measured by the MVO index.

**Trial registration:**

The Microcirculation in Acute Myocardial Infarction (MICRO-AMI). Clinicaltrials.gov NCT01552564. Registered 9th March 2012.

## Background

There has been continued advancement in treatment strategies for ST segment elevation myocardial infarction (STEMI), however, the morbidity and mortality of these patients remains significant. Despite aggressive strategies to quickly restore coronary blood flow in STEMI with PPCI, cardiomyocyte necrosis still occurs[1] and an associated mortality persists[2]. This injury is in part due to cell death after flow restoration known as ischaemia/reperfusion (IR) injury[3]. Mechanical recanalisation of the culprit artery does not always restore optimal blood flow distal to the lesion, a phenomenon known as “no reflow”, which is associated with increased mortality[4]. This is multifactorial and poorly understood but the distal microcirculation is believed to be key to this phenomenon[5].

The index of microcirculatory resistance (IMR) provides a measurement for microcirculatory function[6] using a thermodilution pressure catheter[6]. IMR has been validated in animal[6, 7] and human studies[8].

IMR assessment in STEMI has previously been investigated[9–12]. Microcirculatory dysfunction has been shown to be associated with reperfusion syndrome[13] following STEMI and poor ST segment resolution on ECG[14]. Following myocardial infarction, the IMR predicts infarct size as measured by creatinine kinase rise, wall motion abnormalities as measured on echocardiography[11, 15], and prognosis[16]. There is evidence of a relationship between IMR and microvascular obstruction (MVO) detected by CMR[17], but this has not previously been quantitated.

CMR is increasingly used to measure surrogate endpoints in clinical trials. CMR of myocardial infarction has been correlated with histology[18]. It can accurately measure infarct size[19], LV volumes[20], myocardial salvage[21], MVO[22] and myocardial oedema. Myocardial infarct size[23], myocardial salvage[24] and MVO[25] have all been shown to be associated with worse outcomes. CMR studies have shown that MVO is associated with larger infarct size[26] and a worse prognosis[25], emphasizing that infarct size and MVO are intimately related. However, all these CMR parameters of myocardial damage can only be assessed after primary percutaneous intervention (PPCI). Invasive indexes obtained at the time of PPCI that could predict myocyte and microvascular damage as assessed by CMR would be instrumental for considering potential adjunctive therapies to minimise injury in the PPCI peri-procedural and early recovery phase.

The aims of this study were to investigate whether an invasive parameter, IMR, assessed at the time of PPCI could predict the extent of MVO in proportion to infarct size (MVO index).

## Methods

Consecutive patients presenting with a suspected STEMI to the Bristol Heart Institute catheter laboratory were approached for inclusion in the study. Patients were verbally assented for participation in the study prior to PPCI, formal written informed consent was sought the day following PPCI. This study was approved by the local ethics committee (NHS Southwest: Cornwall and Plymouth, 11/SW/0346). Clinicaltrials.gov NCT01552564. The study protocol conforms to the ethical guidelines of the 1975 Declaration of Helsinki as reflected in a priori approval by the institution's human research committee. (S1 Protocol.).

Patients were included if they were above 18 years old presenting with cardiac symptoms of > 20 mins chest pain or equivalent and ECG criteria consistent with STEMI. Patients needed to be eligible for PPCI and provided assent and consent for the study. Only patients with Thrombolysis In Myocardial Infarction (TIMI) flow ≤ 1 in the infarct related artery (IRA) at presentation were included. Patients were excluded if they had a known allergy to adenosine or gadolinium, chronic atrial fibrillation, renal impairment with eGFR <30, contraindication to angiography, contraindication to CMR (implanted pacemaker/defibrillator, ferromagnetic metal implant, claustrophobia, too obese for CMR scanner), cardiogenic shock, patients with special communication needs or altered consciousness.

IMR was performed following completion of standard PPCI care. Standard PPCI care at this institution during the study included dual antiplatelet therapy (aspirin, prasugrel), bilvalrudin and thrombectomy. Patients received standard post-infarct medical therapy. Consent for continued inclusion in the study was obtained at 24 hours. CMR was undertaken between day 2–4 following myocardial infarction and was repeated at 3 months. MVO index was calculated from the findings on the CMR at 2–4 days after the acute event.

The primary outcome measure was MVO by CMR at 2–4 days.

The IMR analysis was performed by observers blinded to the CMR data. The CMR analysis was performed by observers blinded to the IMR data. Biochemical markers were measured by an independent laboratory technician, without knowledge of the IMR or CMR data.

### Microcirculation: Invasive assessment with IMR

IMR measurements were performed with a Certus Pressurewire (St Jude Medical), as described by Fearon et al[6]. Following reperfusion and deployment of an intracoronary stent, IMR measurement was performed following intracoronary nitrates. The wire was calibrated outside the body, and equalised at the ostium within the guide catheter. The Certus wire was passed into the distal third of the IRA and care was taken not to displace the position of the wire tip throughout the measurements. Baseline measurements were taken using 3 x 3–5 ml boluses of room temperature saline injected through the guide catheter into the IRA. Maximal hyperaemia was induced using an infusion of intravenous adenosine (140 mcg/kg/min) through a large peripheral vein. Further 3 x 3–5 ml boluses of room temperature saline were injected during maximal hyperaemia. The mean aortic and distal coronary pressures were recorded at maximal hyperaemia. IMR was calculated from the mean transit time of saline bolus at hyperaemia multiplied by the distal coronary pressure[6]. All patients received thrombus aspiration prior to IMR, none received GIIb/IIIa inhibitors.

### Microcirculation: Non-invasive assessment with CMR

CMR imaging was carried out with a 1.5T system (Avanto, Siemens, Germany) with a standard 8-phase array receiver coil.

The imaging protocol included localisers and an axial HASTE stack through the thorax in a transverse plane. 4 chamber and 2 chamber single slice localisers were planned off the HASTE imaging, following on to an 8 slice localiser. Long axis cine imaging was performed next with 4, 3 and 2 chamber orientation.

After obtaining scout images, cine steady-state free precession (SSFP) CMR images were acquired during short breath holds in the short axis, two chamber and four chamber planes; on short-axis images the left ventricle was completely encompassed from the base to the apex acquiring a total of 10–12 images. For T2w-STIR imaging a breath-hold black blood segmented turbo spin echo technique was adopted, using a triple inversion recovery preparation module in order to suppress signal from flowing blood as well as from fat, with surface coil normalisation. First-pass rest perfusion images were obtained in the 3 short axis slices. Care was taken to ensure the LVOT was not included in the basal slice. 60 dynamics were acquired encompassing the first pass of the gadolinium contrast (0.1 ml/kg Gadovist 2.0, Bayer Shering Pharma at 4 ml/ second). 10 minutes following contrast administration, a full stack of LGE images were obtained. For LGE imaging, a standard inversion recovery gradient echo sequence was adopted. Acquisition planes were identical to that of T2w STIR and were acquired at least 10 minutes following administration of 0.1 ml/kg Gadovist. The inversion time was progressively optimized to null normal myocardium (typical values, 250–350 ms). Images were acquired on short axis planes covering the entire left ventricle. Each slice was obtained during a breath-hold of 10 to 15s depending on the patient’s heart rate.

### Image analysis

Image analysis for LV volume, function and mass were performed using a semi-automated software (Siemens Medical Solutions, Germany). Endocardial and epicardial borders were delineated manually and propagated to the end systolic and end diastolic frames. Slices were included as ventricular if at least 50% of the slice contained LV myocardium. Papillary muscles were excluded from the endocardial border and included in the ventricular blood pool. Myocardial oedema, infarct size and microvascular obstruction quantitative analyses were performed by manually planimetering the area of abnormal signal intensity using a commercially available semi-automated software (Argus, Siemens) and expressed as a % of LV volume, as previously described[27]. The optimal window setting was defined as the sum of the mean myocardial signal intensity (SI) of the unaffected area plus 2 standard deviations (SD) for this area. The level setting was set at the mean SI of the unaffected area[28]. Early MVO was measured on the rest first-pass perfusion images, whilst late MVO was assessed in the LGE images, which were also used to assess infarct size.

### Statistical analysis

The sample size was calculated for the primary analysis that examined the association between MVO and IMR. A sample size of 50 patients has 80% power to detect a significant association between MVO at 2–4 days and IMR of 0.40 or more, and 90% power to detect a correlation of 0.45 or more at 5% level of statistical significance (2 sided). These values represent moderate correlations between the 2 measures. In a regression analysis, adjusted for 3 covariates, the study has 80% power to detect a difference in the model R^2^ of 0.1 between a model with just the 3 covariates (base model) and one with 3 covariates plus IMR, assuming the base model R^2^ is at least 0.3. These values represent conservative estimates of the association between the variables.

Continuous data were summarised using means and standard deviations (or median and interquartile range if the distribution was skewed). Category variables were reported as a frequency and percentage. The association between the primary outcome, MVO index at 2–4 days and IMR was quantified using linear regression. The analysis was adjusted for three covariates–age, gender and time since MI. There was an *a priori* concern that age and gender were confounders. Time to reperfusion was associated with MVO and MVOI, therefore the adjusted analysis also included this factor. Non-linearity of terms were investigated using multivariable fractional polynomials, and interaction terms were also assessed for inclusion. There were no missing data. We also used an IMR cut off of 40 that has been shown to predict prognosis[16], and IMR quartiles to investigate an association between MVO and IMR. The difference between IMR quartiles was assessed using the Kruskal-Wallis test. The difference between MVO in patients with an IMR more or less than 40 was assessed using the Mann-Witney test. Receiver operator curve (ROC) techniques were used to investigate the relationship between IMR values and MVO, and the optimum cut-off for IMR values was selected using the Liu method, which maximises the product of the sensitivity and specificity. The associations between IMR, MVO and infarct size, troponin and ejection fraction (EF) were assessed using Spearman rank correlation coefficients.

## Results

**Fig 1:**
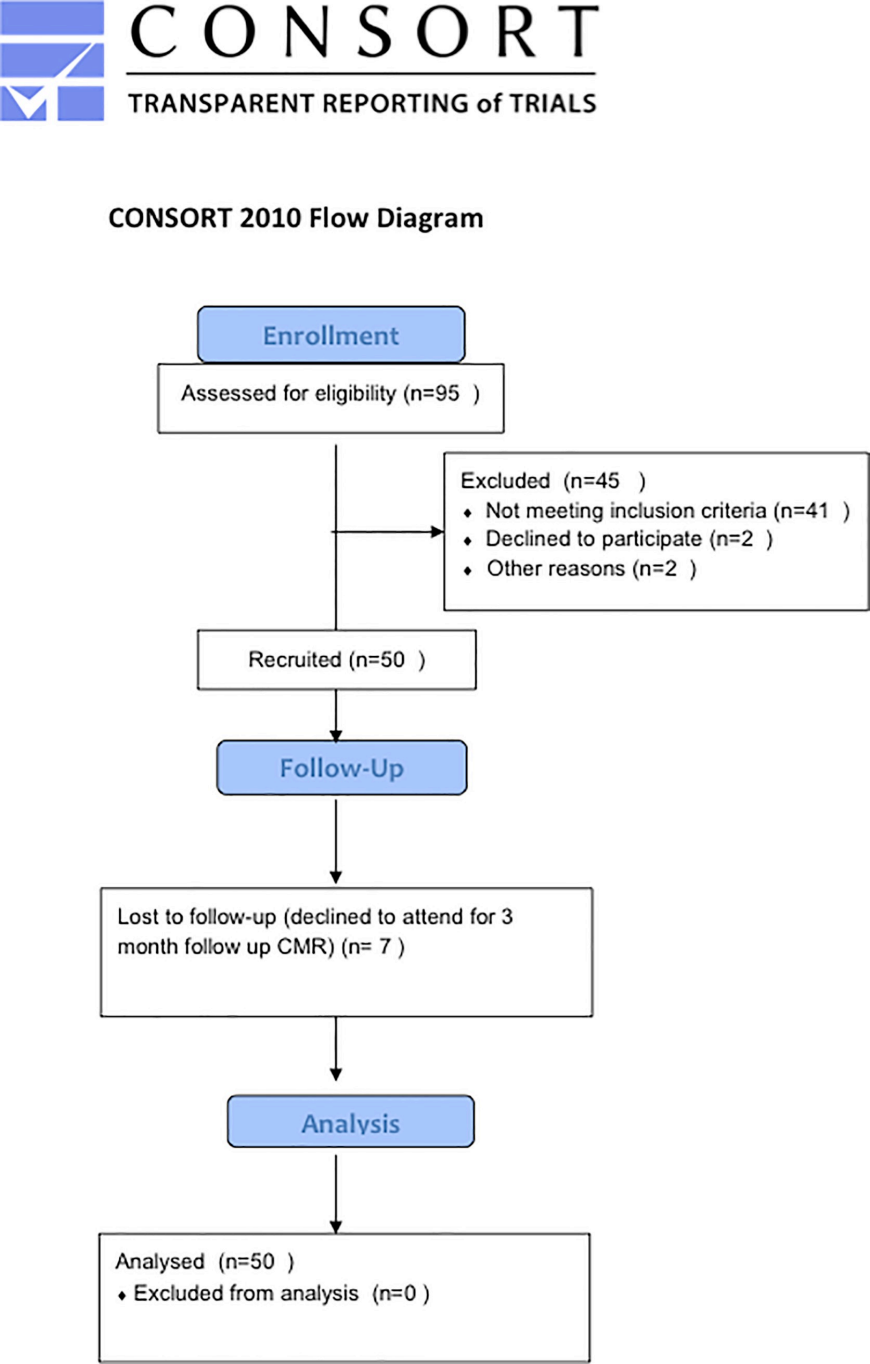
CONSORT diagram.

95 patients were approached for inclusion in the study (Fig 1, S1 Fig) from May 2012 to September 2013. 41 patients were ineligible at the time of PPCI as there was > TIMI 1 flow at presentation. 2 patients were ineligible post PPCI; 1 had intracerebral clips so could not proceed with CMR, and 1 failed to complete IMR measurement due to a complication of PPCI (coronary dissection). 2 patients did not wish to continue with the study following IMR measurement. 50 patients were recruited to the study and completed both baseline IMR and CMR. 7 patients were lost to follow-up. Late MVO occurred in 37 (74%) patients. Baseline and CMR characteristics are shown in Table 1. Baseline characteristics stratified according to IMR cutoff are shown in Table 2.

**Table 1:** Baseline and CMR characteristics.

Median Time MI to CMR (days)	2 (range 1–5)
Age (years)	59.5 (range 38–82)
Sex	12% (6/50) female
	88% (44/50) male
Median time pain to flow restored (minutes)	195 (range 79–882)
Risk Factors:	
Smoking	28% (14/50) ex smokers
	32% (16/50) current smokers
	40% (20/50) non smokers
Positive family history	48% (24/50)
Hypercholesterolaemia	34% (17/50)
Hypertension	38% (19/50)
Previous MI	4% (2/50)
Previous PCI	2% (1/50)
Diabetes	12% (6/50)
Medication at discharge	
Aspirin	10% (5/50)
ACE I	24% (12/50)
Beta blocker	6% (3/50)
Calcium channel blocker	6% (3/50)
Nitrates	0% (0/50)
Statin	24% (12/50)
Nicorandil	0% (0/50)
Angiotensin II inhibitor	4% (2/50)
Prodromal Angina in preceding week	36% (18/50)
IRA:	
LAD	38% (19/50)
RCA	50% (25/50)
LCx	12% (6/50)
TIMI flow in IRA pre PPCI:	
0	96% (48/50)
1	4% (2/50)
TIMI flow in IRA post PPCI:	
2	6% (3/50)
3	94% (47/50)
Median Troponin T (ng/L)	4818 (range 158–10000)
Mean EF at baseline (%)	50 (range 26–66)
Infarct size (% LV *±* SD)	22 *±* 10
Late microvascular obstruction (% LV, median and range)	2.5 (0 to 27.5)
Microvascular obstruction index (median and range)	0.10 (0 to 0.58)

**Table 2:** Baseline characteristics stratified according to IMR cutoff (<40/>40).

IMR	< 40	>40	P value
Age in years (median and range))	55 (39–82)	60 (38–82)	0.23
Sex	Female 19% (5/27)	Female 4% (1/23)	0.2
	Male 81% (22/27)	Male 96% (22/23)	
Median Time pain to flow restored (minutes)	150 (79–492)	238 (104–882)	0.02
IRA:			0.93
LAD	37% (10/27)	39% (9/23)	
RCA	51% (14/27)	48% (11/23)	
LCx	11% (3/27)	13% (3/23)	
TIMI flow in IRA pre PPCI:			0.99
0			
1	96% (26/27)	96% (22/23)	
	4% (1/27)	4% (1/23)	
TIMI flow in IRA post PPCI:			0.09
2	0% (0/27)	13% (3/23)	
3	100% (27/27)	87% (20/23)	
Median Troponin T (ng/L)	3395	5070	0.13

### Primary outcome measure

**Fig 2:**
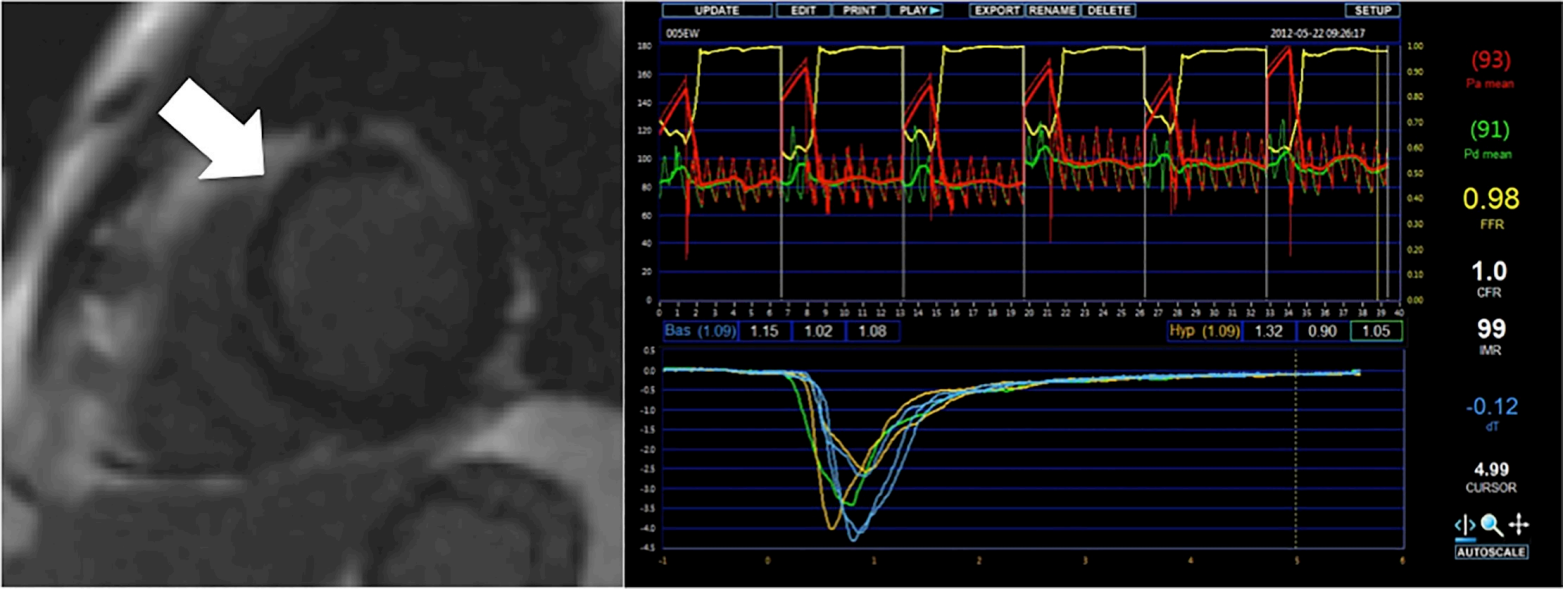
Image of patient with significant late MVO in LAD territory STEMI and corresponding IMR data.

The median IMR was 38.5 (range 9 to 202). The median size of late MVO was 1.9% of the LV (range 0 to 21.0%). Fig 2 demonstrates a patient with significant MVO on CMR and the corresponding IMR data (IMR 99). TIMI III flow was achieved in 94% patients. The mean infarct size measured by CMR was 22% of the LV.

**Fig 3:**
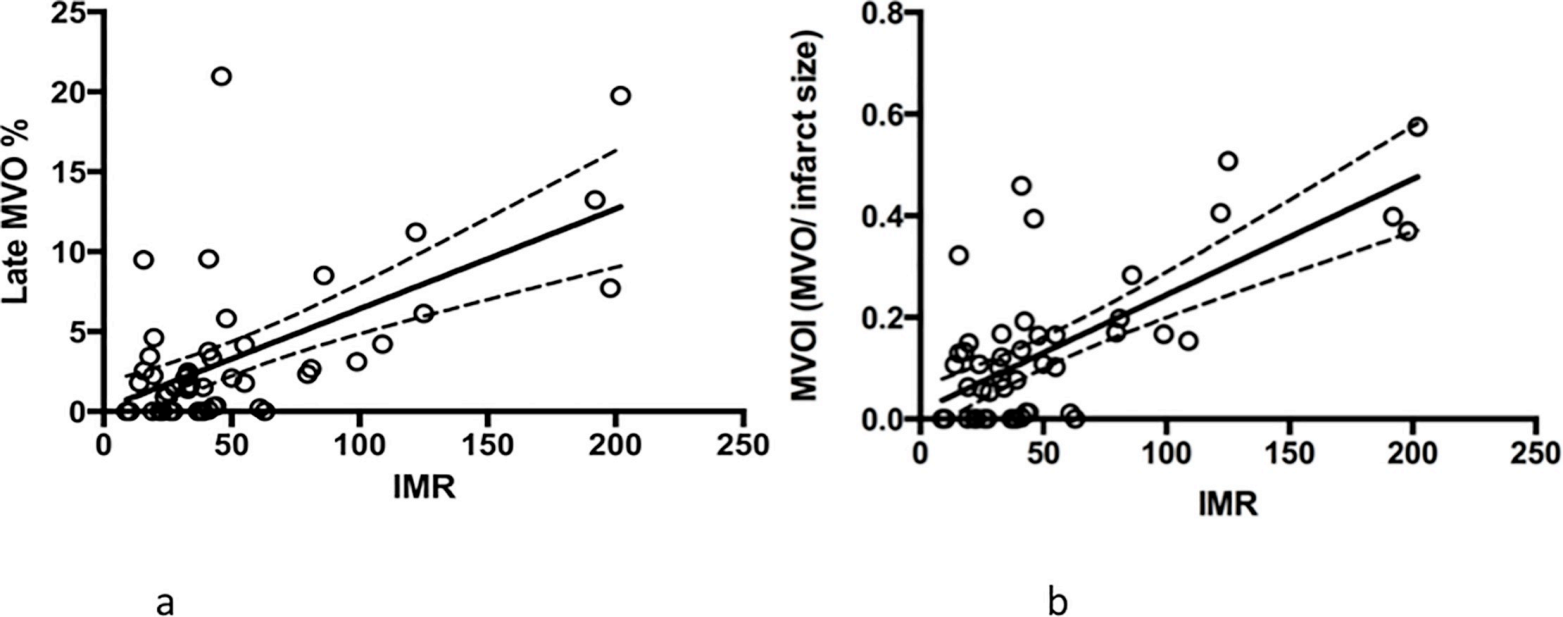
**(a) Unadjusted linear regression of late MVO vs IMR.** Late MVO is significantly associated with IMR (r = 0.611, p<0.001). **(b) Linear regression of MVOI (MVO/ infarct size) vs IMR.** MVOI is significantly associated with IMR (r = 0.702, p<0.001).

By unadjusted linear regression, late MVO is significantly associated with IMR (r = 0.61, [95% CI 0.40, 0.76] p<0.001)(Fig 3A) and the association was even stronger with MVO index (r = 0.70, [95% CI 0.53, 0.82], p<0.001) (Fig 3B. Both associations remained significant (r^2^ = 0.46, p<0.01 for MVO and r^2^ = 0.56, p<0.001 for MVO index) even when adjusted for age, sex and time to reperfusion.

**Fig 4:**
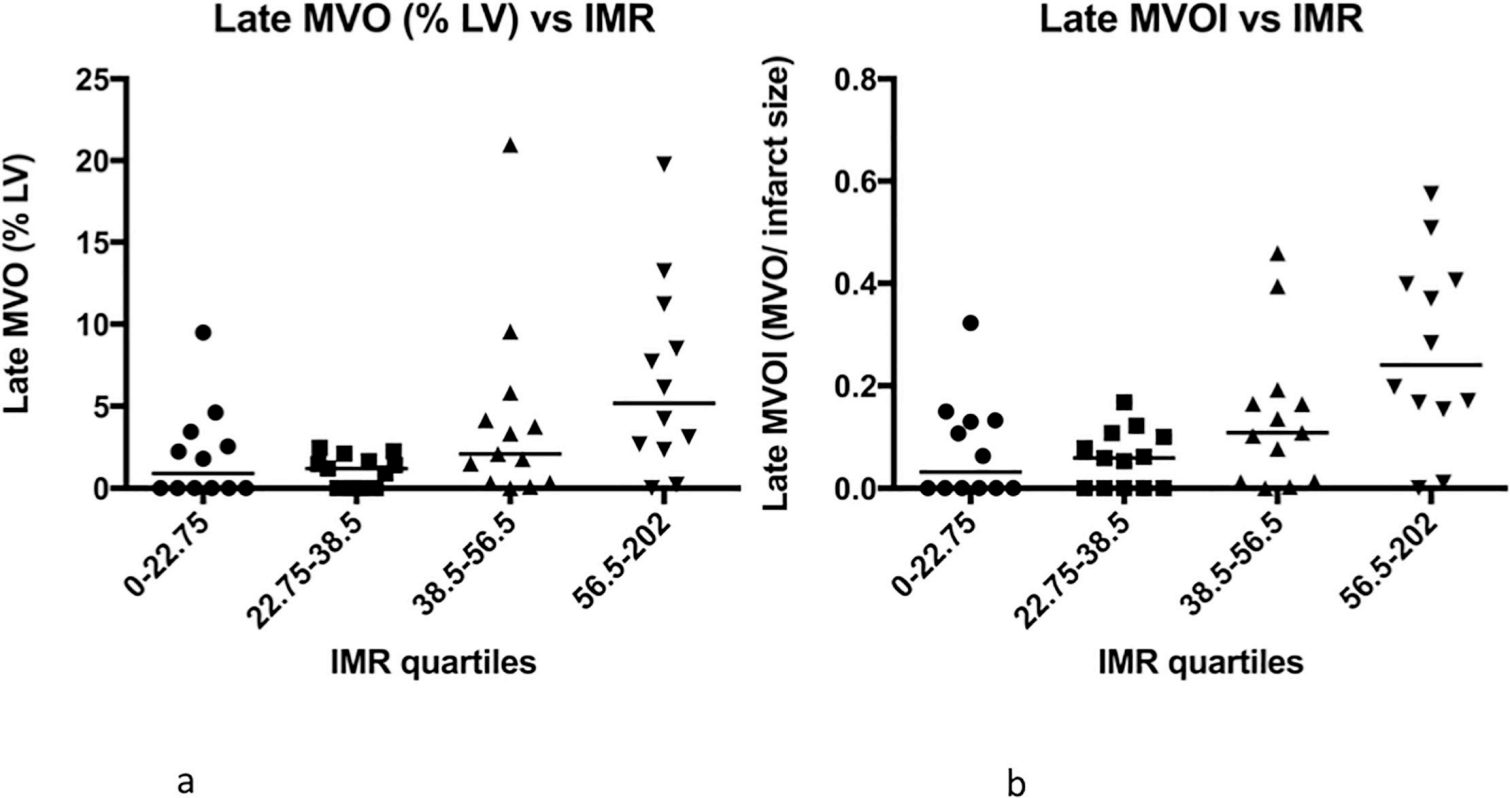
**(a) The association of Late MVO with IMR quartile.** There is a significant difference in IMR with late MVO quartiles (p = 0.006). **(b) The association of Late MVO indexed to infarct size (Late MVOI) with IMR quartiles.** There is a significant difference in IMR with MVOI quartiles (p = 0.002).

On analysis of IMR by quartiles, there is a significant difference in both late MVO (p = 0.006) Fig 4A and MVO index (p = 0.002) Fig 4B. Again, the difference is more marked for MVO index than late MVO alone.

The association between IMR and MVO was stronger in late MVO (more severe myocardial damage) than early MVO (less severe myocardial damage) (p = 0.002 and p = 0.02, respectively).

The ability of IMR to predict late MVO provides an area under the curve (AUC) of 0.78 (95% CI 0.64, 0.92). The optimal IMR cut-off for predicting late MVO is 40, with a sensitivity of 0.59 and 1-specificity of 0.92 (AUC 0.76 at this point). When using an IMR value of 40, this value significantly predicted the presence and degree of late MVO on CMR (p<0.001).

IMR was not associated with infarct size as a % LV calculated from infarct size at baseline (r = 0.04; p = 0.32) nor at 3 months (r = 0.17; p = 0.28), nor to infarct shrinkage (reduction of infarct size at 3 months; r = 0.11; p = 0.49). Late MVO is associated with infarct size by troponin, (r = 0.49; p<0.001) infarct size by CMR at baseline (r = 0.37; p = 0.001) and 3 months (r = 0.40; p = 0.001), and EF at 3 months (r = -0.59; p<0.001).

## Discussion

Our study confirms the hypothesis that the index of microcirculatory resistance (IMR) can be predictive of infarct damage as a composite of microvascular damage over infarct size.

Recent studies support the association of IMR with MVO following STEMI[10, 12]. Our study builds on this further by 1) quantifying the association rather than reporting just the MVO presence/absence alone, as in all previous studies, and by 2) providing a more accurate measurement of MVO as an index of total infarct size.

The mechanism behind microcirculatory dysfunction is multifactorial[5], including distal vessel embolisation[29], vasoconstriction with inflammatory markers[30], external compression from oedema[31]. This study has determined that IMR can predict myocardial damage as a composite of myocardial and microvascular damage, whilst the focus of previous studies was only on the association between IMR and MVO. The results of this study contribute in providing evidence that an invasive parameter such as IMR could potentially identify patients at risk of severe myocardial injury (late MVO) at the time of PPCI, thereby facilitating the delivery of tailored therapy to minimize ischaemia reperfusion injury[32], rather than simply identifying injury with CMR in the recovery phase.

A previous study by Fearon suggests that an IMR cut off following STEMI of > 40 is prognostically significant[16]. In keeping with this data, our data suggests that an IMR cut-off of 40 is predictive of late MVO by CMR. There is a statistically significant difference between the amount of late MVO in patients with and IMR >/ < 40.

In contrast to previous studies[17], IMR is not associated with EF and LV volumes at baseline nor at 3 months. This could be explained by the small sample size and that very few of our patients had a low EF following reperfused STEMI (mean LVEF 50%). Also, time to reperfusion was shorter in our patients compared to previous cohorts. This has also led to a smaller infarct size and smaller size of MVO. The IRA also is important due to region in jeopardy from the occluded coronary artery, ie a high IMR in a proximal LAD lesion would have a different effect on EF, volumes and prognosis than a lesion in a small diagonal branch.

Previous CMR studies have identified late MVO to be associated with infarct size[25, 26]. The results of this study are consistent with late MVO being associated with infarct size by troponin, infarct size at baseline and 3 months by CMR, and EF at 3 months. In fact, a previous study from De Waha et al[33], has demonstrated in a large cohort of STEMI patients that the extent of MVO was only weakly correlated with infarct size. On the other hand a more comprehensive parameter such as MVO index is a more powerful predictor of outcome than infarct size or MVO alone[33]. This was confirmed by our study using an invasive measure of microvascular dysfunction (IMR).

## Study limitations

This is a small single centre study on clinical patients using a single vendor platform for CMR (Siemens). Our study was not powered for clinical outcome and no prognostic data is available for the cohort. Results should be confirmed in larger clinical studies with hard clinical endpoints of prognosis.

Standardisation of IMR measurements was as optimal as possible, however, these were all emergency patients, therefore had not withheld from caffeine. This may have affected response to adenosine and maximal hyperaemia could not be guaranteed in all patients. In addition, there are a proportion of patients who do not respond to adenosine[34, 35]. However, these limitations are not unique to this study and indeed applicable to all previously published IMR studies in the context of STEMI.

IMR was measured at time of PPCI but the late MVO was measured at day 2. Whilst this might have reduced the ability of the IMR to predict late MVO, the timings of these measurements were consistent with previously published studies.

## Conclusions

IMR measured at the time of PPCI in acutely reperfused STEMI is associated with the presence and severity of infarct damage as measured by the MVO index.

## Clinical implication

MVO index is a more sensitive marker for assessing microvascular damage than MVO alone. IMR is a novel, promising, invasive measurement to assess myocardial damage. IMR may allow early selection of patients (at the time of PPCI) that would benefit from novel adjunctive therapies or regenerative stem cell therapies.

## Supporting information

S1 Protocol MicroAMI protocol V7. (PDF)

S1 Fig Consort 2010 checklist. (DOC)
