# Supplementary material for: Microvascular dysfunction determines infarct characteristics in patients with reperfused ST-segment elevation myocardial infarction: The MICROcirculation in Acute Myocardial Infarction (MICRO-AMI) study
Source: PLoS One. 2018 Nov 13;13(11):e0203750. doi: 10.1371/journal.pone.0203750 (PMC6233915; doi:10.1371/journal.pone.0203750)
Supplement: S1 Protocol — (PDF) [file pone.0203750.s001.pdf]

The microcirculation in acute myocardial infarction (Micro-AMI)

REC No: 11/SW/0346
ISRCTN No: NCT01552564
R+D no: CS/2011/3854
University of Bristol: Study 1625

Details of Sponsor

Dr Birgit Whitman
Head of Research Governance, University of Bristol
Quality Assurance Lead
Research & Enterprise Development (RED)
Senate House, Tyndall Avenue, Bristol BS8 1TH
Tel: +44 (0) 117 331 7130 (ext. 17130)
Mob: +44 (0) 7787 154834
Fax: +44 (0) 117 929 8383
e-mail: Birgit.Whitman@bristol.ac.uk

Chief Investigators & Research Team Contact Details

Chief Investigator
Dr Elisa McAlindon
Research Fellow
School of Clinical Sciences
University of Bristol
Level 7, Bristol Royal Infirmary
Queen's Building
Bristol BS2 8HW

Tel: 0117 3422526
Email: elisa.mcalindon@bristol.ac.uk

Co-PI
Dr Chiara Bucciarelli Ducci
Senior Lecturer and Consultant Cardiologist
School of Clinical Sciences
University of Bristol
Level 7, Bristol Royal Infirmary
Queen's Building
Bristol BS2 8HW

Tel: 0117 3423145
Email: c.bucciarelli-ducci@bristol.ac.uk

Co-PI
Andreas Baumbach
Cardiology Consultant
Bristol Heart Institute
Bristol
BS2 8HW
Tel:
Email: *andreas.baumbach@uhbristol.ac.uk*

Table of contents

Glossary / abbreviations **4**
1. Trial summary **5**
2. Background **5**
3. Aims and objectives **7**
4. Plan of Investigation **8**
4.1 Trial schema 8
4.2 Trial design 8
4.3 Trial population 8
4.4 Primary and secondary outcomes 9
4.5 Sample size calculation 10
5. Trial methods **10**
5.1 Research procedures 10
5.2 Duration of treatment period 10
5.3 Definition of end of trial 11
5.4 Data collection 11
5.5 Planned recruitment rate 12
5.6 Participant recruitment 12
5.7 Discontinuation/withdrawal of participants 12
5.8 Frequency and duration of follow up 13
5.9 Likely rate of loss to follow-up 13
5.10 Expenses 13
5.11 Measures taken to avoid bias 13
6. Statistical analyses **13**
6.1 Plan of analysis 13
6.2 Frequency of analyses 14
7. Trial management **14**
7.1 Day-to-day management 14
7.2 Monitoring of sites 14
7.3 Trial Steering Committee and Data Monitoring and Safety Committee 14
8. Safety reporting **15**
8.1 Expected adverse events 15
8.2 Period for recording serious adverse events 17
9. Ethical considerations **17**
9.1 Review by an NHS Research Ethics Committee 17
9.2 Risks and anticipated benefits 18
9.3 Informing potential study participants of possible benefits and known risks 18
9.4 Obtaining informed consent from participants 18
10. Research governance **18**
10.1 Sponsor approval 18
10.2 NHS approval 18
10.3 Investigators' responsibilities 19
10.4 Monitoring by sponsor 19
10.5 Indemnity 19
11. Data protection and participant confidentiality **19**
11.1 Data protection 19
11.2 Data handling, storage and sharing 19
12. Dissemination of findings **20**

13. Amendments to protocol 20
14. References 21

Glossary / abbreviations

ACS	Acute coronary syndrome
AE	Adverse event - any undesirable event in a subject receiving treatment according to the protocol, including occurrences which are not necessarily caused by or related to administration of the research procedures.
AR	Adverse reaction – any undesirable experience that has happened a subject while taking a drug that is suspected to be caused by the drug or drugs
BHI	Bristol Heart Institute
BRI	Bristol Royal Infirmary
BRU	Biomedical Research Unit
CABG	Coronary artery bypass grafting
CAD	Coronary artery disease
CMR	Cardiac magnetic resonance
CRB	Cardiovascular research board
CRF	Case report form
CTEU	Clinical Trials and Evaluation Unit
DMSC	Data monitoring and safety committee
ECG	Graphical representation of electrical activity of the heart over time, as recorded by an electrocardiograph
eGFR	Estimated glomerular filtration rate: derived from gender, age, ethnicity and serum creatinine
IMR	Index of microcirculatory resistance
IS	Infarct size
LV	Left ventricle
MACE	Major cardiac adverse event
MI	Myocardial infarction
MVO	Microvascular resistance
NIHR	National Institute for Health Research
PCI	Percutaneous coronary intervention
PPCI	Primary percutaneous intervention
PIL	Patient information leaflet
REC	Research ethics committee
SAE	Serious adverse event - events which result in death, are life threatening, require hospitalisation or prolongation of hospitalisation, result in persistent or significant disability or incapacity.
SAR	Serious adverse reaction
SOP	Standard operating procedure
SSAR	Suspected serious adverse reaction
STEMI	ST segment elevation myocardial infarction
SUSAR	Suspected unexpected serious adverse reaction - an untoward medical occurrence suspected to be related to a medicinal product that is not consistent with the applicable product information and is serious.
TMG	Trial management group
TSC	Trial steering committee
UH Bristol	University Hospitals Bristol NHS Foundation Trust

1. Trial summary

This is a single centre study in heart attack patients. This study investigates the relationship between the narrowing in small heart artery vessels as measured by a technique called the index of microcirculatory resistance and the effects of this narrowing as measured on cardiac magnetic resonance imaging.

2. Background

Summary for the Non-Expert

- Coronary heart disease (CHD) is a leading cause of death in the UK. During a heart attack (myocardial infarction) a blockage in a coronary artery prevents the flow of oxygen and nutrients to the territory of the heart muscle supplied by this artery resulting in damage to the heart. Some of this damage is reversible, some irreversible. Following a heart attack, the extent of the damaged heart muscle increases the risk of developing chronic heart failure.
- The treatment for a heart attack is two-fold: 1) medication to inhibit clot formation and reduce risks of further coronary events and medication to prevent the development of heart failure in the longer term; and 2) mechanical stabilisation of the culprit lesion in the coronary artery with percutaneous coronary intervention (PCI) and intracoronary stent deployment.
- Patients suffering heart attacks present a variety of different heart attacks size and characteristics as assessed by cardiovascular MRI. The determinants for these variable phenotypes are not complete clear and explored.
- Despite a successful PCI, a significant amount of damage still occurs to the heart muscle. This research proposal investigates the association between the degree of microvascular resistance using the index of microvascular dysfunction (IMR), and the myocardial infarct characteristics on cardiac magnetic resonance imaging (CMR). By quantitating the association between IMR and the myocardial infarct characteristics on cardiac magnetic resonance imaging, this study aims to develop clinical endpoints that can be used to assess the benefit of future strategies to minimise ischaemia/ reperfusion injury and thus to minimise damage caused by a myocardial infarction.
- Patients with heart attacks will be recruited via the Bristol Heart Institute.
- The multidisciplinary team has the required skills and experience for undertaking this clinical study and will include: cardiac MRI and Interventional cardiologists, cardiac biology specialists clinical Trials Unit expertise (Clinical Trials Manager, Statisticians, Database manager).The costs of the study will be for staffing, IMR catheters, cardiac MRI scans and clinical trial unit costs. There will be no costs to the NHS.

RATIONALE FOR CURRENT STUDY

Coronary heart disease (CHD) causes over 90,000 deaths a year in the UK: approximately one in five deaths in men and one in six deaths in women. Death rates from CHD in the UK are still amongst the highest in Western Europe. CHD is not only the single most common cause of death in the UK; it is also very costly, imposing a huge annual burden on UK economy. Despite continued advancement in treatment strategies for myocardial infarctions, the morbidity and mortality of patients presenting with myocardial infarction remains significant.

Novel insights into the microcirculatory effects of a myocardial infarction and infarct characteristics may offer new strategies to tailor therapy to improve health outcomes. By quantitating the association between IMR and the myocardial infarct characteristics on cardiac magnetic resonance imaging, this study aims to develop clinical endpoints that can be used to assess the benefit of future strategies to minimise ischaemia/ reperfusion injury and thus to minimise damage caused by a myocardial infarction. This study will also identify the patients at a higher risk following STEMI who would benefit the most from early adjuvant therapies as they become available.

Despite aggressive strategies to restore coronary blood flow quickly in an acute myocardial infarction with PPCI, cardiomyocyte necrosis still occurs[1] and an associated mortality persists[2]. This injury is in part due to cell death after flow restoration; the result of a biochemical signalling cascade known as ischaemia/reperfusion (IR) injury. Mechanical opening of the culprit artery does not always ensure good blood flow distal to the lesion; a condition referred to as "no reflow" which is associated with an increased mortality[3]. This is multifactorial and poorly understood but the distal microcirculation is believed to be key to this phenomenon.

The index of microcirculatory resistance (IMR) can be measured using a using thermodilution pressure catheter. The fractional flow reserve (FFR), the coronary flow reserve (CFR) and IMR can be measured simultaneously. The feasibility and reliability of thermodilution-derived CFR has previously been established[4]. The IMR specifically assesses the microvascular disease. IMR has been validated in animal[5] and human studies[6] and these studies have demonstrated that IMR measurement is largely independent of variations in hemodynamic state[7] and therefore superior to FFR and CFR as it is not impacted by stenosis in the epicardial vessel[8,9].

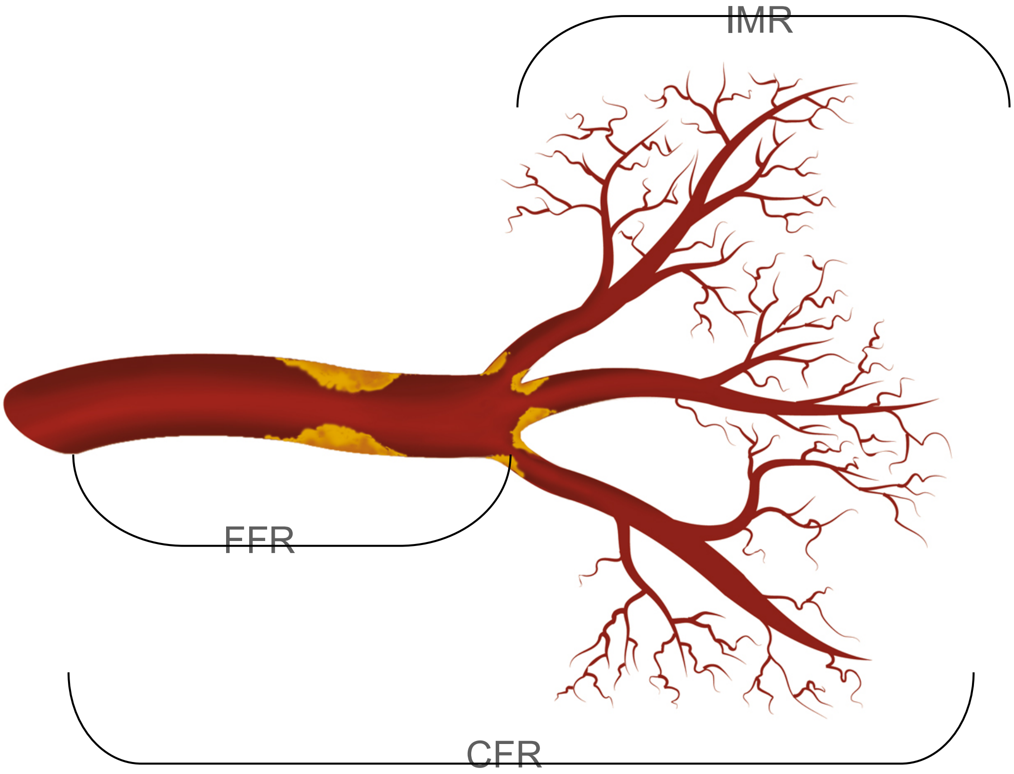

Proof of concept studies for Doppler flow measurements in acute myocardial infarction have previously been carried out[10]. Microcirculatory dysfunction has been shown to be associated

with reperfusion syndrome[11] following acute myocardial infarction and poor ST segment resolution on ECG[12]. Following myocardial infarction, the IMR predicts infarct size as measured by creatinine kinase rise and wall motion abnormalities as measured on echocardiography[13]. The degree of microcirculatory dysfunction following STEMI as measured by the index of microcirculatory resistance (IMR) is correlated with both infarct size as measured by troponin[14], presence or absence of MVO[15] on CMR and can predict LV function on transthoracic echocardiogram following STEMI[16].

CMR has become the gold standard for surrogate endpoints in clinical trials. CMR of myocardial infarction has been correlated with histology[17]. It can accurately measure infarct size[18], LV volumes[19], myocardial salvage[20], microvascular obstruction (MVO)[21] and myocardial oedema[14]. Myocardial infarct size[22], myocardial salvage[23] and MVO[24] have all been shown to be associated with worse outcomes. CMR studies have shown that MVO is associated with larger infarct size[25] and a worse prognosis[26].

Circulating micro-RNA have recently been identified as novel biomarkers in cardiovascular disease[27]. Levels of circulating micro-RNA are increased in acute coronary syndromes and myocardial infarction[28-36]. Circulating micro-RNA have been validated against troponin levels[30,32,33,27] and may provide insight into the cause of ischaemia/ reperfusion injury[37-42] and consequently the no reflow phenomenon.

All the factors that lead to a large myocardial infarction, and consequently a worse outcome, are not known. This study aims to identify myocardial infarctions that are associated with microcirculatory dysfunction and the CMR infarct characteristic of poor prognosis. By quantitating the association between IMR and the myocardial infarct characteristics on cardiac magnetic resonance imaging, we aim to validate this technique to risk stratify patients with myocardial infarction and therefore identify patients who would benefit from future interventional treatments to minimize damage caused by a myocardial infarction.

A search of registry databases suggests there are no ongoing studies examining the associations between the index of microcirculatory resistance and infarct characteristics on CMR.

3. Aims and objectives

Aims:
To quantitate the association between IMR and MI phenotype.
To investigate the association between biomarkers and IMR with known CMR characteristics of poor outcomes.

Research Question:
In ST segment elevation myocardial infarction (STEMI) patients undergoing primary percutaneous coronary intervention (PPCI), does the degree of microcirculatory dysfunction assessed by the index of microcirculatory dysfunction (IMR) predict the phenotype of myocardial infarction as evaluated by cardiac magnetic resonance (CMR)?

4. Plan of Investigation

4.1 Trial schema

Myocardial Infarction

↓

STEMI presenting via PPCI service to BHI

↓

Verbal assent in catheterisation lab

↓

PPCI including IMR
Biomarkers

↓

Formal consent day 1
Biomarkers

↓

CMR baseline day 2

↓

CMR follow up 3 months
Biomarkers

Figure 1 Trial schema

4.2 Trial design

Single centre study investigating the association between infarct characteristics and microcirculatory dysfunction based at the Bristol Heart Institute. Microcirculatory dysfunction will be assessed using the index of microcirculatory resistance (IMR). Data collection and analysis will be undertaken in the Bristol Heart Institute NIHR BRU.

4.3 Trial population

- **Study population:**

Male and female patients (>18 years old) presenting with a suspected STEMI (ST-elevation on electrocardiogram) within 12 hours of chest pain. No coronary collaterals and TIMI flow <1.

• **Planned interventions:**
STEMI patients intervention as part of clinical care: On arrival to hospital, emergency patients with STEMI will be taken directly to the cardiac catheterisation for primary PCI. This is time critical with national targets of door to balloon time of 60 mins. Standard primary PCI treatment includes thromboaspiration and restoration of flow with stent deployment.

STEMI patients additional measurement as part of research study: Following completion of standard treatment of the heart attack, the IMR measurement will be performed; a pressure thermistor wire will be passed through the catheter already in situ for the PPCI. To achieve maximal hyperaemia for this measurement, an infusion of adenosine will be administered via a peripheral cannula. Verbal assent for inclusion in the study and this measurement will be performed on arrival in the catheter lab following the explanation of the PPCI. This is in accordance with previous and current studies in the department (Comfortable, Pinpoint, Erupt).

All acute coronary syndrome medical treatment will be unaffected. Consent for continued inclusion in the study will be performed at 24 hours.

At day 2-4 following myocardial infarction, baseline CMR will be undertaken. This will be repeated at 3 months. Blood samples for biomarkers will be taken at the time of angioplasty, at 24 hours and at the 3 months CMR follow up visit.

4.3.1 Inclusion criteria

Participant may enter study if ALL of the following apply
- >18 years old
- Cardiac symptoms of > 20 mins chest pain or equivalent.
- ECG criteria consistent with STEMI (STEMI)
- Proceeding with percutaneous angioplasty
- Assent/ consent to the study

4.3.2 Exclusion criteria

Participant may not enter study if ANY of the following apply
- Known allergy to adenosine or gadolinium
- Chronic atrial fibrillation
- Renal impairment with eGFR <30
- Contraindication to angiography
- Contraindication to CMR (implanted pacemaker/ defibrillator, ferromagnetic metal implant/ injury, claustrophobia, obesity/ too large for CMR scanner)
- Cardiogenic shock
- Patients with special communication needs or altered consciousness.
- Patients who do not give assent/consent to the study

4.4 Primary and secondary outcomes

4.4.1 Primary outcome

Microvascular obstruction as a percentage of the left ventricle at 2-4 days.

.

4.4.2 Secondary outcomes

CMR outcomes: Myocardial infarct size, microvascular obstruction, myocardial salvage, LV volumes, mass and ejection fraction at baseline and at 3 months follow up CMR.
Biomarker outcomes: micro-RNA and proteomics on admission, 24 hours and at follow up
Angiographic outcomes: TIMI score in the infarct related artery pre and post PPCI, myocardial blush grade following PPCI, BARI and APPROACH scores following PPCI, coronary collateral circulation (Rentrop classification)
ECG: ST segment resolution at 90 minutes following PPCI

4.5 Sample size calculation

The sample size has been calculated for the primary analysis which will examine the association between MVO and IMR.
The study size has been set at 50 patients. A study of this size will have 80% power to detect a significant correlation between MVO at 2-4 days and IMR of 0.40 or more and 90% power to detect a correlation of 0.45 or more, at 5% statistical significance (2-sided). These values represent moderate correlations between the two measures. In a regression analysis, adjusted for three covariates, the study will also have 80% power to detect a difference in the model R^2 of 0.1 between a model with just the three covariates (base model) and one with the three covariates plus IMR, assuming the base model R^2 is at least 0.3. These values represent conservative estimates of the association between the variables. If the base model R^2 is greater 0.3 then the power will increase and smaller changes will be detectable with 80% power. For example, if the base model R^2 is assumed to be 0.7, a difference in model R^2 of 0.05 could be detected with 80% power.

5. Trial methods

On arrival to hospital, patients will be assented for IMR during their primary PCI. Following thromboaspiration, restoration of flow, and deployment of intracoronary stent. IMR will be performed following completion of standard PPCI care. All acute coronary syndrome medical treatment will be unaffected. Consent for continued inclusion in the study will be performed at 24 hours. At day 2-4 following myocardial infarction, baseline CMR will be undertaken. This will be repeated at 3 months. Blood samples for biomarkers will be taken at the time of angioplasty, at 24 hours and at the 3 months CMR follow up visit.

5.1 Research procedures

In addition to standard care for STEMI, patients will have IMR measured following stent deployment in the catheter lab. They will also have 2 CMR scans; 1 at baseline (day 2-4) and 1 at 3 months follow-up. Blood samples for biomarkers will be taken at baseline, 24 hours and 3 months.

5.2 Duration of treatment period

Patients will be included in the study until their 3 month follow up CMR is completed.

5.3 Definition of end of trial

End of trial will be confirmed when the final patient has completed the 3 month follow up CMR.

5.4 Data collection

Data collection will include the following elements:

(a) A log of STEMI patients who are approached for the study (including the date when they are given the Patient Information Leaflet (PIL)).

(b) Patients approached and assessed against the eligibility criteria.

(c) Assent, consent and baseline information (history, ECG, troponin and angiogram results) collected.

(d) CMR scan data

(e) IMR data

(f) Biomarker results

Exam	Time scale				
	Pre-treatment	During PPCI	Day 1	Day 2	Month 3
MRI				X	X
Assent	X				
Informed consent			X		
IMR		X			
Blood test for biomarkers		X	X		X

Table 1 Data collection

5.5 Planned recruitment rate

We plan to recruit 50 consecutive patients who meet the inclusion/ exclusion criteria.
BHI admits > 600 STEMI patients a year. We expect recruitment to last 3 months (estimated 11 STEMI patients a week, we estimate one third of these will be eligible for recruitment).

5.6 Participant recruitment

Patients presenting with STEMI and meeting inclusion/ exclusion criteria will be invited to participate. Potential trial participants will be identified in the catheter lab as they present with STEMI through the PPCI service. A verbal explanation of the planned trial will be given and verbal assent to be included taken. This is in accordance with previous and current studies running in the department (Comfortable, Erupt, Pinpoint). Following PPCI, the patients will be transferred to the coronary care unit. All participants will be given a PIL (approved by the local Research Ethics Committee (REC)) describing the study. The patient will have time to read the PIL and to discuss their continuation in the study with others outside the research team (e.g. relatives or friends) if they wish. Most patients will have at least 6 hours to consider whether to participate. Despite the short notice, it is important to include these patients as the changes on CMR following STEMI are time critical and waiting longer would not be possible.

After admission to the coronary care unit following their STEMI, patients will be seen by a member of the local research team (study clinician/research nurse/trial co-ordinator) who will answer any questions, confirm the patient’s eligibility and take written informed consent if the patient decides to continue in the study. Details of all patients approached for the trial and reason(s) for non-participation (e.g. reason for being ineligible or patient refusal) will be documented.

5.7 Discontinuation/withdrawal of participants

Participants/ clinicians may withdraw from the trial by contacting a member of the research team. If IMR data and CMR scans have been completed, the data will still be collected and included in the data analysis.

If a participant wishes to withdraw, we will continue to analyse any data already collected, unless the participant expresses a wish for their samples and any associated data to be destroyed.

5.8 Frequency and duration of follow up

In addition to standard PPCI out patient clinic follow up, patients will be followed up at 3 months with CMR and biomarker blood testing.

5.9 Likely rate of loss to follow-up

Until discharge from hospital, the only losses to follow-up will be due to death or a participant withdrawing; these losses are expected to be very few. We expect loss to 3 month follow-up to be minimal.

5.10 Expenses

Reimbursement of travel expenses for the 3 month follow up CMR visit will be offered.

5.11 Measures taken to avoid bias

This is a non randomised trial enrolling consecutive consenting eligible STEMI patients.

The IMR analysis will be performed by observers blinded to the CMR data.

The CMR analysis will be performed by observers blinded to the IMR data.

Biochemical markers will be measured by an independent laboratory technician, without knowledge of IMR or CMR data.

6. Statistical analyses

6.1 Plan of analysis

Continuous data will be summarised using means and standard deviations (or median and interquartile range if the distribution is skewed). Category variables will be reported at a frequency and percentage. The association between the primary outcome, MVO at 2-4 days and IMR will quantified using multiple linear regression, assuming the measures will be transformed (e.g. to a logarithmic scale) as necessary to induce normality. The analysis will be adjusted for three covariates – age, gender and time since MI. The relationship between the outcome and each predictor will be explored graphically and the model will be extended to include non-linear functions if non-linear relationships are indicated.
Secondary outcomes will also be analysed using multiple linear regression (continuous measures) or logistic regression (binary measures). Continuous outcomes measured at both 2-4 days and at 3 months will be modelled using a mixed regression model, which will account for the correlation between repeated measurements on the same individual. If a baseline value is also available this will be measured jointly with the subsequent measures.
Associations will be quantified and reported with 95% confidence intervals.

6.2 Frequency of analyses

The primary analysis will take place when follow-up is complete for all recruited participants. No formal interim analysis is planned.

7. Trial management

The trial will be managed by the Trial Management Group (TMG). The TMG will prepare all the trial documentation and data collection forms, develop and maintain the study database, check data quality as the trial progresses, monitor recruitment and carry out trial analyses with advice from the Clinical Trials and Evaluation Unit (CTEU) Bristol.

7.1 Day-to-day management

The trial will be managed by a Trial Management Group (TMG), which will meet approximately monthly. The TMG will be chaired by a Chief Investigator and will include all members of the named research team (see *Chief Investigators & Research Team Contact Details)*.

An appropriately qualified person by training will be responsible for identifying potential trial participants, seeking informed participant consent, collecting trial data and ensuring the trial protocol is adhered to.

7.2 Monitoring of sites

7.2.1 Initiation visit

Before the study commences training session(s) will be organised by TMG. These sessions will ensure that personnel involved fully understand the protocol, CRFs and the practical procedures for the study.

7.2.2 Site monitoring

The study will be monitored in line with the sponsors monitoring policy.

7.3 Trial Steering Committee and Data Monitoring and Safety Committee

The Cardiovascular Research Board (CRB) has been established to oversee cardiovascular projects being carried out by the partnership between Hospitals Bristol NHS Foundation Trust (UH Bristol) and the University of Bristol (primarily, component projects of National Institute for Health Research (NIHR) Applied Programme Grants, the NIHR Cardiovascular Biomedical Research Unit (BRU)). The CRB is made up of representatives of UH Bristol and the University of Bristol. The Board will act as the Trial Steering Committee for this and other single centre trials in these NIHR programmes. The Board is chaired by the Director of the BRU, Professor Angelini.

.

8. Safety reporting

Serious and other adverse events will be recorded and reported in accordance with the International Conference for Harmonisation of Good Clinical Practice (ICH GCP) guidelines and the Sponsor's Research Related Adverse Event Reporting Policy (see Figure 2). The UH Bristol perform this role on behalf of the University of Bristol.

CMR is a safe imaging technique with no ionising radiation. There is a small risk due to contrast administration. These side effects are not unexpected. The research team will only notify fatal and 'unexpected' non-fatal SAEs to the Trial Sponsor. Unexpected events are those not listed in the trial protocol or on the CRFs. The sponsor will inform the TMG if the event needs to be reported to the REC (usually only events that are both related to the intervention and unexpected e.g. SUSARs see Figure 2).

8.1 Expected adverse events

The following adverse events are 'expected':

Inability to complete the scan due to claustrophobia

Side effects due to contrast administration

	Adverse reactions from clinical trial data (experience in more than 2900 patients)		Additional adverse reactions from postmarketing spontaneous reporting
**System Organ Class**	**Uncommon**	**Rare**	**Rare**
**Immune system disorders**		Anaphylactoid reaction	Anaphylactoid shock
**Nervous system disorders**	Headache,<br>Dizziness,<br>Paraesthesia,<br>Dysgeusia	Parosmia	Loss of consciousness,<br>Convulsion
**Eye disorders**			Conjunctivitis,<br>Eyelid oedema
**Cardiac disorders**			Cardiac arrest,<br>Tachycardia
**Vascular disorders**	Vasodilation	Hypotension	Circulatory collapse,<br>Flushing
**Respiratory, thoracic and mediastinal disorders**		Dyspnoea	Respiratory arrest,<br>Bronchospasm,<br>Cyanosis,<br>Oropharyngeal swelling,<br>Cough,<br>Sneezing
**Gastrointestinal disorders**	Nausea	Vomiting	
**Skin and subcutaneous tissue disorders**		Urticaria,<br>Rash	Face oedema,<br>Hyperhidrosis,<br>Pruritus,<br>Erythema
**General disorders and administration site conditions**	Injection site pain,<br>Injection site reaction		Nephrogenic Systemic Fibrosis (NSF),<br>Feeling hot,<br>Malaise

Perioperative MI, including:
Raised Troponin T or Troponin I
Cardiac arrest, requiring:

- Resuscitation involving ventricular defibrillation/DC shock
- Chest reopening
- External/internal cardiac massage

Haemodynamic support, including use of:

- Any inotropes
- IABP

- Pulmonary artery catheter
- Vasodilator
- Low cardiac output, requiring management with a Swan-Ganz catheter, an intra-aortic balloon pump, or left ventricular assist device

Arrythmias, including:

- Supraventricular tachycardia or atrial fibrillation requiring treatment
- VF/VT requiring intervention
- Pacing

Figure 2 Serious adverse event reporting flow chart

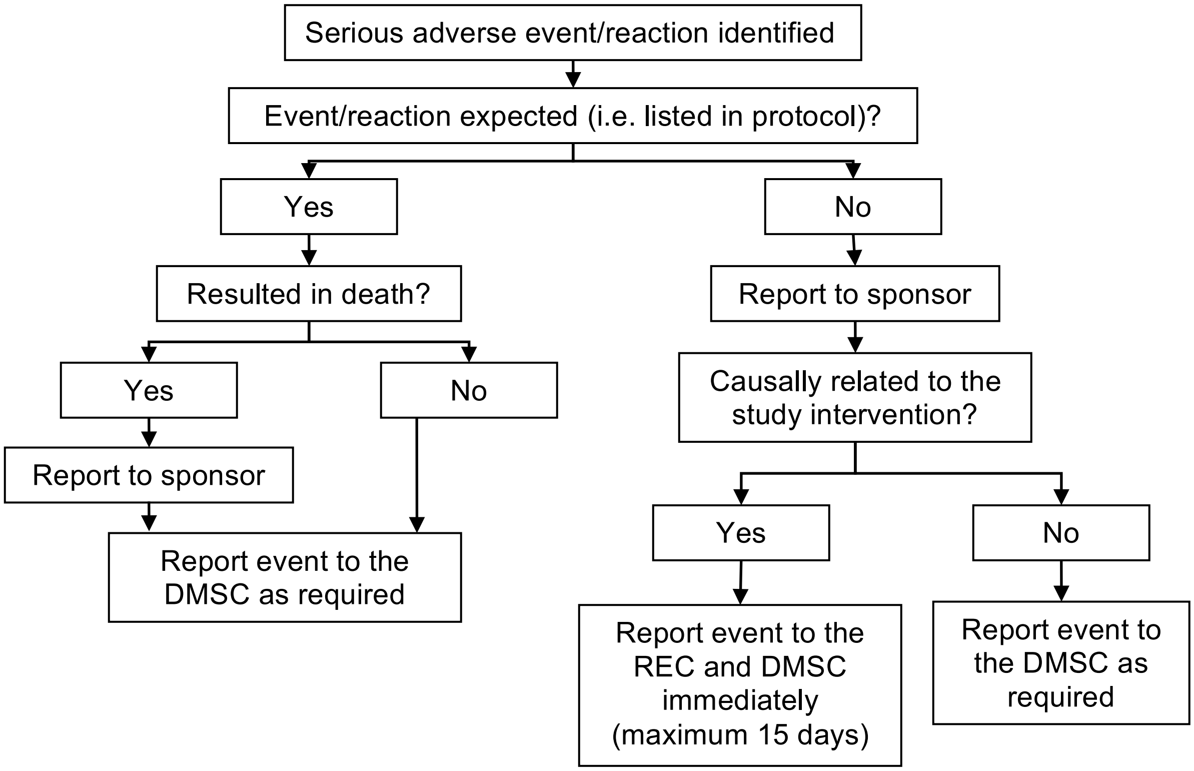

8.2 Period for recording serious adverse events

Data on adverse events will be collected from the time of assent to the end of the second scan.

9. Ethical considerations

9.1 Review by an NHS Research Ethics Committee

Ethics review of the protocol for the trial and other trial related essential documents (e.g. PIL and consent form) will be carried out by a UK Research Ethics Committee (REC).

Any amendments to these documents, after a favourable opinion from the REC has been given, will be submitted to the REC for approval prior to implementation.

9.2 Risks and anticipated benefits

Potential benefits to participants: CMR clinical report will be generated and available to clinical care team with assessment of LV function and infarct characteristics that may guide future management.

Potential harms to participants: There is a small risk of side effects due to contrast administration during CMR. There is a negligible incremental risk of IMR measurement above those for PPCI.

Benefits to society: this study will allow better understanding of STEMI with potential benefits to future patients.

9.3 Informing potential study participants of possible benefits and known risks

Information about possible benefits and risks of participation will be described in the PIL.

9.4 Obtaining informed consent from participants

All participants will be required to give written informed consent. This process, including the information about the trial given to patients in advance of recruitment, is described above in section 5.7.

The research nurse/trial coordinator/PI/clinical research fellow will be responsible for the consent process, which will be described in detail in the Trial Manual.

10. Research governance

This study will be conducted in accordance with:

- International Conference for Harmonisation of Good Clinical Practice (ICH GCP) guidelines
- Research Governance Framework for Health and Social Care

10.1 Sponsor approval

Any amendments to the trial documents must be approved by the sponsor prior to submission to the REC.

10.2 NHS approval

Approval from the local NHS Trust is required prior to the start of the trial.

Any amendments to the trial documents approved the REC will be submitted to the Trust for information or approval as required.

10.3 Investigators' responsibilities

Investigators will be required to ensure that local research approvals have been obtained and that any contractual agreements required have been signed off by all parties before recruiting any participant. Investigators will be required to ensure compliance to the protocol and study manual and with completion of the CRFs. Investigators will be required to allow access to study documentation or source data on request for monitoring visits and audits performed by the Sponsor or any regulatory authorities.

Investigators will be required to read, acknowledge and inform their trial team of any amendments to the trial documents approved the REC that they receive and ensure that the changes are complied with.

10.4 Monitoring by sponsor

The study will be monitored and audited in accordance with the Sponsor's policy, which is consistent with the Research Governance Framework. All study related documents will be made available on request for monitoring and audit by the sponsor, the relevant REC and for inspection by the MHRA or other licensing bodies.

10.5 Indemnity

The University of Bristol is the sponsor for this research study and will arrange insurance.

11. Data protection and participant confidentiality

11.1 Data protection

Data will be collected and retained in accordance with the UK Data Protection Act 1998.

11.2 Data handling, storage and sharing

11.2.1 Data handling

Data will be entered onto a purposed designed database and data validation and cleaning will be carried out throughout the trial. Standard operating procedures (SOPs) for database use, data validation and data cleaning will be available and regularly maintained.

11.2.2 Data storage

All study documentation will be retained in a secure location during the conduct of the study and for 5 (non-CTIMP) /15 (CTIMP) years after the end of the study, when all patient identifiable paper records will be destroyed by confidential means. Prior to destruction, paper records will be scanned and stored on the University server with limited password controlled access. Where trial related information is documented in the medical records, these records will be identified by a label bearing the name and duration of the trial in accordance to UHBristol policy. In compliance with the MRC Policy on Data Preservation, relevant 'meta'-data about the trial and the full dataset, but without any participant identifiers other than the unique participant identifier, will be held indefinitely (University server). A secure electronic 'key' with a unique participant identifier, and key personal identifiers (.e.g. name, date of birth and NHS number)

will also be held indefinitely, but in a separate file and in a physically different location (NHS hospital server). These will be retained because of the potential for the raw data to be used subsequently for secondary research.

11.2.3 Data sharing

Data will not be made available for sharing until after publication of the main results of the study. Thereafter, anonymised individual patient data will be made available for secondary research, conditional on assurance from the secondary researcher that the proposed use of the data is compliant with the MRC Policy on Data Preservation and Sharing regarding scientific quality, ethical requirements and value for money. A minimum requirement with respect to scientific quality will be a publicly available pre-specified protocol describing the purpose, methods and analysis of the secondary research, e.g. a protocol for a Cochrane systematic review. The second file containing patient identifiers would be made available for record linkage or a similar purpose, subject to confirmation that the secondary research protocol has been approved by a UK REC or other similar, approved ethics review body.

12. Dissemination of findings

The findings will be disseminated by usual academic channels, i.e. presentation at international meetings, as well as by peer-reviewed publications and through patient organisations and newsletters to patients, where available.

13. Amendments to protocol

Amendment number (i.e. REC and/or MHRA amendment number)	Previous version	Previous date	New version	New date	Brief summary of change	Date of ethical approval (or NA if non-substantial)
Minor Am 1	V5	28/11/2011	NA	NA	Date extension 1/12/13	NA
SA2	V5	28/11/2011	V6	13/5/13	Addition proteomics to biomarkers	5/6/13

14. References

[1] Ginks WR, Sybers HD, Maroko PR, Covell JW, Sobel BE, Ross J, Jr. Coronary artery reperfusion. II. Reduction of myocardial infarct size at 1 week after the coronary occlusion. J. Clin. Invest. October 1972 1972;51(10):2717-2723.

[2] Keeley EC, Boura JA, Grines CL. Primary angioplasty versus intravenous thrombolytic therapy for acute myocardial infarction: a quantitative review of 23 randomised trials. Lancet. Jan 4 2003;361(9351):13-20.

[3] Valgimigli M, Campo G, Malagutti P, Anselmi M, Bolognese L, Ribichini F, Boccuzzi G, de Cesare N, Rodriguez AE, Russo F, Moreno R, Biondi-Zoccai G, Penzo C, Díaz Fernández JF, Parrinello G, Ferrari R. Persistent coronary no flow after wire insertion is an early and readily available mortality risk factor despite successful mechanical intervention in acute myocardial infarction: a pooled analysis from the STRATEGY (Single High-Dose Bolus Tirofiban and Sirolimus-Eluting Stent Versus Abciximab and Bare-Metal Stent in Acute Myocardial Infarction) and MULTISTRATEGY (Multicenter Evaluation of Single High-Dose Bolus Tirofiban Versus Abciximab With Sirolimus-Eluting Stent or Bare-Metal Stent in Acute Myocardial Infarction Study) trials.
JACC Cardiovasc Interv. 2011;4(1):51-62.

[4] Barbato E, Aarnoudse W, Aengevaeren WR, Werner G, Klauss V, Bojara W, Herzfeld I, Oldroyd KG, Pijls NH, De Bruyne B. Validation of coronary flow reserve measurements by thermodilution in clinical practice. Eur Heart J.; 2004:219-223.

[5] Fearon WF, Balsam LB, Farouque HM et al. Novel index for invasively assessing the coronary microcirculation. Circulation. United States, 2003:3129-32.

[6] Ng MK, Yeung AC, Fearon WF. Invasive assessment of the coronary microcirculation: Superior reproducibility and less hemodynamic dependence of index of microcirculatory resistance compared with coronary flow reserve. Circulation. 2006:2054-2061.

[7] Ng MK, Yeung AC, Fearon WF. Invasive assessment of the coronary microcirculation: Superior reproducibility and less hemodynamic dependence of index of microcirculatory resistance compared with coronary flow reserve. Circulation. 2006:2054-2061.

[8] Fearon WF, Aarnoudse W, Pijls NH et al. Microvascular resistance is not influenced by epicardial coronary artery stenosis severity: experimental validation. Circulation. United States, 2004:2269-72.

[9] Aarnoudse W, Fearon WF, Manoharan G et al. Epicardial stenosis severity does not affect minimal microcirculatory resistance. Circulation. United States, 2004:2137-42.

[10] Hirsch A, Nijveldt R, Haeck JD et al. Relation between the assessment of microvascular injury by cardiovascular magnetic resonance and coronary Doppler flow velocity measurements in patients with acute anterior wall myocardial infarction. J Am Coll Cardiol. United States, 2008:2230-8.

[11] Feldman LJ, Himbert D, Juliard JM et al. Reperfusion syndrome: relationship of coronary blood flow reserve to left ventricular function and infarct size. J Am Coll Cardiol. United States, 2000:1162-9.

[12] Feldman LJ, Coste P, Furber A et al. Incomplete resolution of ST-segment elevation is a marker of transient microcirculatory dysfunction after stenting for acute myocardial infarction. Circulation. United States, 2003:2684-9.

[13] Fearon WF, Shah M, Ng M et al. Predictive value of the index of microcirculatory resistance in patients with ST-segment elevation myocardial infarction. J Am Coll Cardiol. United States, 2008:560-5.

[14] Kitabata H, Imanishi T, Kubo T et al. Coronary microvascular resistance index immediately after primary percutaneous coronary intervention as a predictor of the transmural extent of infarction in patients with ST-segment elevation anterior acute myocardial infarction. JACC Cardiovasc Imaging. United States, 2009:263-72.

[15] McGeoch R, Watkins S, Berry C, Steedman T, Davie A, Byrne J, Hillis S, Lindsay M, Robb S, Dargie H, Oldroyd K.the index of microcirculatory resistance measured acutely predicts the extent and severity of myocardial infarction in patients with ST segment elevation myocardial infarction. J Am Coll Cardiol Cardiovasc Interv. 2010 Jul;3(7):715-22.

[16] McGeoch R, Watkins S, Berry C, Steedman T, Davie A, Byrne J, Hillis S, Lindsay M, Robb S, Dargie H, Oldroyd K.the index of microcirculatory resistance measured acutely predicts the extent and severity of myocardial infarction in patients with ST segment elevation myocardial infarction. J Am Coll Cardiol Cardiovasc Interv. 2010 Jul;3(7):715-22.

[17]Kim R, Fieno D, Parrish T, Harris K, Chen E, Simonetti O, Bundy J, Finn J, Klocke F, Judd R. Relationship of MRI delayed contrast enhancement to irreversible injury, infarct age, and contractile function, Circ 1999; **100**(19):1992–2002.

[18] Mahrholdt H, Wagner A, Holly T, Elliott MD, Bonow R, Kim R, Judd R. Reproducibility of chronic infarct size measurement by contrast-enhanced magnetic resonance imaging. Circ 2002; **106**:2322–2327

[19] Danilouchkine M, Westernberg J, De Roos A, Reiber J, Lelieveldt B. Operator induced variability in cardiovascular MR: left ventricular measurements and their reproducibility. J Cardiovasc Magn Res 2005; 7: 447–457

[20] Desch S, Engelhardt H, Meissner J, Eitel I, Sareban M, Fuernau G, de Waha S, Grothoff M, Gutberlet M, Schuler G, Thiele H. Reliability of myocardial salvage assessment by cardiac magnetic resonance imaging in acute reperfused myocardial infarction. Int J Cardiovasc Imaging. 2011 Jan 30.

[21] Mather AN, Lockie T, Nagel E, Marber M, Perera D, Redwood S, Radjenovic A, Saha A, Greenwood JP, Plein S. Appearance of microvascular obstruction on high resolution first-pass perfusion, early and late gadolinium enhancement CMR in patients with acute myocardial infarction. J Cardiovasc Magn Reson. 2009;11:33.

[22] Bello D, Einhorn A, Kaushal R, Kenchaiah S, Raney A, Fieno D, Narula J, Goldberger J, Shivkumar K, Subacius H, Kadish A. Cardiac magnetic resonance imaging: infarct size is an independent predictor of mortality in patients with coronary artery disease. Magn Reson Imaging. 2011 Jan;29(1):50-6.

[23] Eitel I, Desch S, Fuernau G, Hildebrand L, Gutberlet M, Schuler G, Thiele H. Prognostic significance and determinants of myocardial salvage assessed by cardiovascular magnetic resonance in acute reperfused myocardial infarction. Am Coll Cardiol. 2010;55(22):2470-9.

[24] Cochet A, Lorgis L, Lalande A. Major prognostic impact of persistent microvascular obstruction as assessed by contrast-enhanced cardiac magnetic resonance in reperfused acute myocardial infarction. Eur Radiol 2009, 19:2117-26.

[25] de Waha S, Desch S, Eitel I, Fuernau G, Zachrau J, Leuschner A, Gutberlet M, Schuler G, Thiele H. Impact of early vs. late microvascular obstruction assessed by magnetic resonance imaging on long-term outcome after ST-elevation myocardial infarction: a comparison with traditional prognostic markers. Eur Heart J. 2010;31(21):2660-8.

[26] Cochet AA, Lorgis L, Lalande A. Major prognostic impact of persistent microvascular obstruction as assessed by contrast-enhanced cardiac magnetic resonance in reperfused acute myocardial infarction. Eur Radiol 2009; 19:2117-26.

[27] Corsten MF, Dennert R, Jochems S et al. Circulating MicroRNA-208b and MicroRNA-499 reflect myocardial damage in cardiovascular disease. Circ Cardiovasc Genet. United States, 2010:499-506.

[28] Ai J, Zhang R, Li Y et al. Circulating microRNA-1 as a potential novel biomarker for acute myocardial infarction. Biochem Biophys Res Commun. United States: 2009 Elsevier Inc, 2010:73-7.

[29] Cheng Y, Tan N, Yang J et al. A translational study of circulating cell-free microRNA-1 in acute myocardial infarction. Clin Sci (Lond). England, 2010:87-95.

[30] D'Alessandra Y, Devanna P, Limana F et al. Circulating microRNAs are new and sensitive biomarkers of myocardial infarction. Eur Heart J. England, 2010:2765-73.

[31] D'Alessandra Y, Pompilio G, Capogrossi MC. Letter by D'Alessandra et al regarding article, "Circulating microRNA-208b and microRNA-499 reflect myocardial damage in cardiovascular disease". Circ Cardiovasc Genet. United States, 2011:e7; author reply e8.

[32] Gidlof O, Andersson P, van der Pals J, Gotberg M, Erlinge D. Cardiospecific microRNA Plasma Levels Correlate with Troponin and Cardiac Function in Patients with ST Elevation Myocardial Infarction, Are Selectively Dependent on Renal Elimination, and Can Be Detected in Urine Samples. Cardiology. Switzerland: Basel., 2011:217-26.

[33] Meder B, Keller A, Vogel B et al. MicroRNA signatures in total peripheral blood as novel biomarkers for acute myocardial infarction. Basic Res Cardiol 2011;106:13-23.

[34] Wang R, Li N, Zhang Y, Ran Y, Pu J. Circulating MicroRNAs are Promising Novel Biomarkers of Acute Myocardial Infarction. Intern Med. Japan, 2011:1789-95.

[35] Wang GK, Zhu JQ, Zhang JT et al. Circulating microRNA: a novel potential biomarker for early diagnosis of acute myocardial infarction in humans. Eur Heart J. England, 2010:659-66.

[36] Widera C, Gupta SK, Lorenzen JM et al. Diagnostic and prognostic impact of six circulating microRNAs in acute coronary syndrome. J Mol Cell Cardiol: 2011 Elsevier Ltd, 2011.

[37] He B, Xiao J, Ren AJ et al. Role of miR-1 and miR-133a in myocardial ischemic postconditioning. J Biomed Sci. England, 2011:22.

[38] Heyn J, Hinske C, Mohnle P, Luchting B, Beiras-Fernandez A, Kreth S. MicroRNAs as Potential Therapeutic Agents in the Treatment of Myocardial Infarction. Curr Vasc Pharmacol, 2011.

[39] Kukreja RC, Yin C, Salloum FN. MicroRNA - New Players in Cardiac Injury and Protection. Mol Pharmacol, 2011.

[40] Salloum FN, Yin C, Kukreja RC. Role of microRNAs in cardiac preconditioning. J Cardiovasc Pharmacol 2010;56:581-8.

[41] Squadrito F, Deodato B, Squadrito G et al. Gene transfer of IkappaBalpha limits infarct size in a mouse model of myocardial ischemia-reperfusion injury. Lab Invest 2003;83:1097-104.

[42] Ye Y, Perez-Polo JR, Qian J, Birnbaum Y. The role of microRNA in modulating myocardial ischemia-reperfusion injury. Physiol Genomics. United States, 2011:534-42.
